# Description of OXA-244 carbapenemase-producing *Escherichia coli* in farm animals in The Netherlands, 2024

**DOI:** 10.1093/jac/dkag155

**Published:** 2026-05-15

**Authors:** K T Veldman, A van Essen-Zandbergen, Y Geurts, F Harders, B Wit, J A Stegeman, A P A Hendrickx, M S M Brouwer, Dutch CPE Surveillance Study Group, A Jansz, A Ott, A Troelstra, A E Muller, A J van Griethuysen, A L E van Arkel, A L M Vlek, A P van Dam, B Maraha, B Zwart, B M W Diederen, C Oliveira dos Santos, D C Melles, D W van Dam, E Bathoorn, E de Jong, E Kolwijck, E van der Vorm, E I G B de Brauwer, F Koene, H Berkhout, J da Silva, J de Vries, J Rahamat-Langendoen, J C Sinnige, J R Lo Ten Foe, J W Dorigo-Zetsma, J W T Cohen Stuart, K van Dijk, K Waar, M de Graaf, M den Reijer, M van Rijn, M Wong, M A Leversteijn-van Hall, M P A van Meer, M P M van der Linden, N Al Naiemi, P Gruteke, R Steingrover, R van Mansfeld, S Dinant, S Paltansing, S B Debast, S J Vainio, S P van Mens, T Schulin, W Ang, W van den Bijllaardt

**Affiliations:** Wageningen Bioveterinary Research, Wageningen University & Research, Houtribweg 39, Lelystad, the Netherlands; Wageningen Bioveterinary Research, Wageningen University & Research, Houtribweg 39, Lelystad, the Netherlands; Wageningen Bioveterinary Research, Wageningen University & Research, Houtribweg 39, Lelystad, the Netherlands; Wageningen Bioveterinary Research, Wageningen University & Research, Houtribweg 39, Lelystad, the Netherlands; Netherlands Food and Consumer Product Safety Authority (NVWA), Food Safety, Utrecht, The Netherlands; Faculty of Veterinary Medicine, Utrecht University, Yalelaan 7, Utrecht, the Netherlands; National Institute for Public Health and the Environment (RIVM), Centre for Infectious Disease Control, Bilthoven, The Netherlands; Wageningen Bioveterinary Research, Wageningen University & Research, Houtribweg 39, Lelystad, the Netherlands; Institute for Risk Assessment Sciences (IRAS), Faculty of Veterinary Medicine, Utrecht University, Yalelaan 2, Utrecht, the Netherlands

## Abstract

**Background:**

To minimize the risk of livestock becoming a reservoir of carbapenemase-producing Enterobacterales (CPE), it is prohibited to use carbapenems in food-producing animals in Europe. Nonetheless, CPE have been detected in several EU countries in pigs, poultry and cattle.

**Objectives:**

To detect and characterize CPE from farm animals in the Netherlands obtained within the European monitoring programme for antimicrobial resistance in animals and food.

**Methods:**

Caecal samples from livestock animals are screened for the presence of CPE according to the recommended EURL-AR protocol and supplementary molecular screening. WGS was used to further study the isolates and compare with human-derived CPE.

**Results:**

In 2024, two CPE isolates were detected in caecal samples from a pig and a broiler. Both were *Escherichia coli* producing OXA-244 and showed reduced susceptibility to meropenem, imipenem and ertapenem. WGS confirmed the presence of *bla*_OXA-244_ gene on a chromosomally located IS-element. Although both isolates belonged to ST58 and were genetically similar, neither represented a clonal relationship with each other, nor with human-associated OXA-244-producing *E. coli* from national CPE surveillance. Follow-up investigations on both farms did not reveal additional CPE.

**Conclusions:**

The finding of two OXA-244-producing *E. coli* isolates in livestock caecal samples demonstrates the added value of implementing a more sensitive screening method for detection of CPE with low-level resistance to carbapenems in the European AMR monitoring programme for animals and food.

## Introduction

Infections caused by carbapenemase-producing Enterobacterales (CPE) pose a growing public health problem due to limited treatment options.^1^ To minimize the risk for development of an animal reservoir of CPE, the use of carbapenem antibiotics in animals is prohibited in the European Union (EU).^2,3^ Until about a decade ago, CPE was rarely found in farm animals in Europe. However, the European Food Safety Authority (EFSA) has recently reported various CPE in farm animals in a growing number of EU countries.^4^ In the period 2022–2023, this included *Escherichia coli* carrying *bla*_OXA-48_ (Spain, Portugal and Romania); *bla*_OXA-181_ (Italy, Spain and Portugal); *bla*_OXA-244_ (Portugal); *bla*_NDM-1_ (Norway), *bla*_NDM-5_ (Czech Republic, Italy and Spain) or *bla*_VIM-1_ (Austria and Denmark). Interestingly, fattening pigs appear to be the most frequent source for CPE compared to cattle and poultry.^4^ CPE had not been found in farm animals or in nationally produced food within the AMR monitoring in the Netherlands until 2024.^5^ Previously, the only known cases of CPE in animals in the Netherlands were reported in dogs.^6^ Here, we describe two recent findings of OXA-244-producing CPE in Dutch farm animals identified through the European monitoring programme for antimicrobial resistance in animals and food. Both isolates were detected using a supplemental protocol in parallel to the EURL-AR recommended protocol for detection of CPE. These livestock- associated isolates were compared with human-retrieved *bla*_OXA-244_ encoding CPE. Across Europe, *bla*_OXA-244_ is increasingly identified in *E. coli* in human patients but no direct genetic link was detected between human and animal isolates.

## Materials and methods

As part of the mandatory AMR programme (Decision EU/2020/1729), faecal samples of livestock are screened for the presence of CPE according to the EURL-AR protocol^7^ based on non-selective enrichment, followed by culturing on chromogenic plates, see annex I (available as Supplementary data at *JAC* Online) for full details. Since 2012, these samples are also screened for the presence of CPE by selective enrichment followed by PCR screening and subsequent culturing of PCR-positive samples, targeting NDM, KPC, VIM, IMP, OXA-48, IMI and FRI carbapenemase genes. PCR-positive samples are cultured on several selective media: MacConkey agar with 0.125 mg/L ertapenem, ChromID^®^ CARBA agar and ChromID^®^ OXA agar plates. After pure culturing on blood agar plates and identification with MALDI-TOF (MALDI Biotyper^®^ Sirius System, Bruker), antimicrobial susceptibility testing is performed using broth microdilution in harmonized European antibiotic panels (EUVSEC3, Sensititre^®^, Thermo Scientific) according to ISO standards.^8^ Results were interpreted with epidemiological cut-off values (ECOFFs) from EUCAST.^9^ WGS was performed using Illumina MiSeq and Oxford Nanopore Technologies MinION (Data available through ENA accession PRJEB106400). Hybrid assembly was performed using Unicycler v0.5.1. Whole-genome multilocus sequence typing (wgMLST) and cluster analysis were performed in context of Dutch CPE surveillance WGS data as described previously, with a maximum cut-off of 25 allelic differences to the nearest neighbour for potential clonal isolates.^10^ Inverse PCR and sequencing experiments were performed to test for the potential mobilization of the *bla*_OXA-244_ gene facilitated by IS elements using outward-directed primers targeting the *bla*_OXA-244_ gene, further described in Annex I.

## Results

### Sampling and bacterial isolation

In 2024, *bla*_OXA-244_ encoding *E. coli* were detected in two caecal samples from farm animals collected at slaughter using selective enrichment followed by PCR-screening and bacterial culturing. The first CPE-suspected isolate (designated: WBVR_CPE_1.47) was identified in June 2024 from a pooled sample of 10 broilers originating from a single flock cultured on MacConkey agar with meropenem. The second CPE-suspected isolate (designated: WBVR_CPE_1.53) was detected in September 2024 from a caecal sample of an individual fattening pig again on MacConkey agar with meropenem. The farms from which these animals originated were located approximately 130 km apart.

### Susceptibility testing and molecular analysis

Both *E. coli* isolates showed reduced susceptibility to all carbapenem antibiotics tested: meropenem (MIC: 0.12–0.25 mg/L, ECOFF 0.06 mg/L); imipenem (MIC: 1 mg/L, ECOFF 0.5 mg/L); and ertapenem (MIC: 0.5 mg/L, ECOFF 0.03 mg/L). Both were resistant to ampicillin (MIC: >32 mg/L, ECOFF 8 mg/L), but remained susceptible to third generation cephalosporins. The broiler isolate (WBVR_CPE_1.47) did not show any additional resistance, whereas the pig isolate (WBVR_CPE_1.53) was resistant to both sulfamethoxazole (MIC: >512 mg/L, ECOFF 64 mg/L) and trimethoprim (MIC: >16 mg/L, ECOFF 2 mg/L).

WGS analysis of WBVR_CPE_1.47 revealed no plasmids or additional resistance genes. WBVR_CPE_1.53 harboured *bla*_TEM-1_, *dfrA*5, *sul*2, *aph(3’)-Ia*, *aph(3'’)-Ib* and *aph(6)-Id* all located on a multi-replicon plasmid containing IncFIB, IncFII and IncQ1. An IncI2 plasmid and a Mob-P plasmid were also present, but did not carry resistance genes.

**Figure 1.**
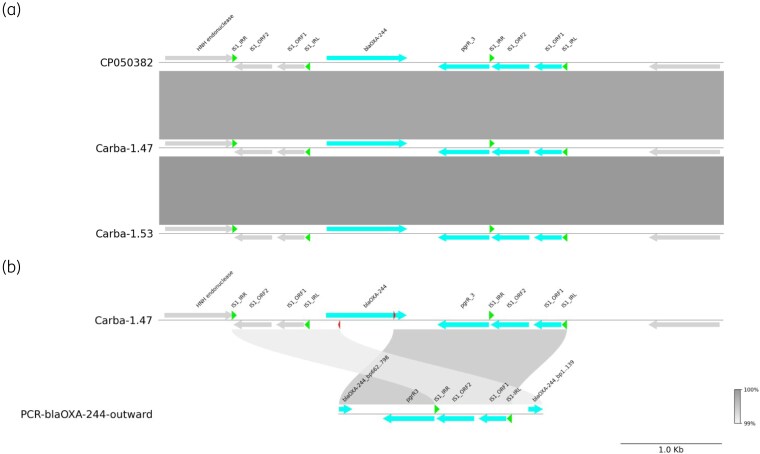
Comparison of genetic sequences flanking *bla*_OXA-244_. (a) Comparison of the chromosomal insertion site of the *bla*_oxa-244_ gene in the detected isolates, compared to reference CP050382. (b) Schematic representation of the PCR product of the detected circular intermediate of the IS*1*-*bla*_OXA-244_ genetic element. Red triangles indicate the location of the primers for the inverse PCR.

**Figure 2.**
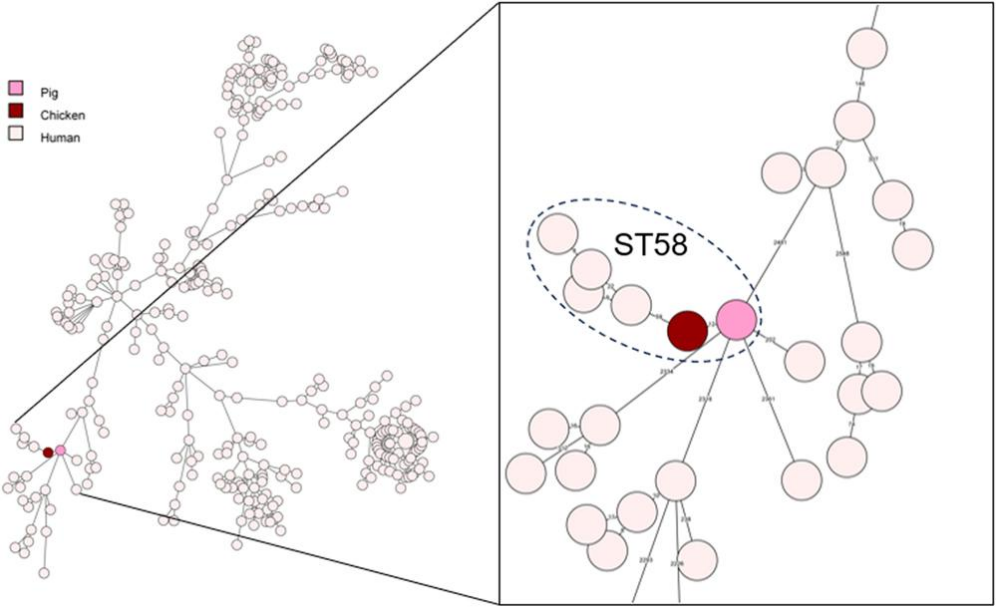
wgMLST comparison of *bla*_OXA-244_ producing *E. coli* isolates from farm animals and humans, sources indicated by colour. The inset depicts the isolates that belong to MLST type ST58.

Both *E. coli* belonged to MLST ST58 and carried *bla*_OXA-244_ on a chromosomally located IS-element. Whole-genome MLST demonstrated that the *E. coli* isolates were genetically related (12 wgMLST alleles difference, 214 core genome SNP differences). Each isolate contains two copies of IS*1* in tandem with *bla*_OXA-244_ in between (Figure 1a). Mobilization of *bla*_OXA-244_ by IS*1* into a small circular element was shown in both isolates using PCR assays with outward facing primers from the *bla*_OXA-244_ gene and sequencing the resulting product (Figure 1b). Comparison of the two animal OXA-244-producing *E. coli* isolates with *n* = 318 genetically highly diverse *bla*_OXA-244_-carrying *E. coli* from humans obtained in the Dutch CPE surveillance between January 2016 and August 2025, revealed no genetic relatedness (Figure 2). The closest related human isolate from ST58 contained 59 wgMLST allele differences from WBVR_CPE_1.47. Additional comparison of the OXA-244-producing *E. coli* to ESBL-encoding *E. coli* ST58 isolates from livestock animals collected in the Netherlands between 2021 and 2024 (*bla*_CTX-M-1_, *bla*_CTX-M-8_, *bla*_CTX-M-15_, *bla*_CTX-M-55_, *bla*_CTX-M-65_, *bla*_SHV-12_, *n* = 96) also revealed no genetic relatedness (greater than 130 allelic differences, data not shown).

## Discussion

Given the current increase in the reporting of CPE in farm animals in Europe^1^ and the outlook in a recent Dutch publication quantifying the risk of introduction of CPE on Dutch livestock farms,^11^ the first report of CPE in livestock in the Netherlands is not unexpected. However, the finding of two genetically related OXA-244-positive *E. coli* isolates detected in different farm animal sectors within a relatively short period is noteworthy and suggests the presence of a shared yet unidentified source. A human reservoir within the Netherlands appears unlikely, since comparative wgMLST analysis of Dutch human and veterinary OXA-244-positive *E. coli* ST58 isolates revealed no direct genetic link.

Following these detections, both farms were voluntarily visited in November 2024 by the Netherlands Food Safety Authority. A structured survey addressing animal health, antibiotic use, feed, farm management and hygiene was conducted, and faecal samples were collected from a representative subset of animals according to the Dutch CPE research plan. No common epidemiological link was found between the two farms, and all samples tested negative for *bla*_OXA-244_, indicating a low risk of onward farm-level transmission.

Across Europe, *bla*_OXA-244_ is increasingly identified in *E. coli* ST38,^12^ ST131^13^ and other sequence types in human patients,^14^ while it is only rarely found in human *E. coli* ST58 in the Netherlands. Transmission of OXA-244-producing *E. coli* isolates via the food chain, the environment or animal contact are considered plausible.^14^ However, in livestock, only a single European case has previously been documented: an *E. coli* carrying *bla*_OXA-244_ detected in a caecal sample of fattening pig from Portugal in 2023, without further details about location of the gene.^1^ Outside Europe, *bla*_OXA-244_ was identified on the chromosome of a single *E. coli* ST10 isolate obtained from a fattening pig in Egypt in 2022.^15^ Still, OXA-244-producing *E. coli* isolates are further spreading in the human community often without knowledge of source and route.^14,16^ Therefore, it is most relevant to continue and improve the screening for CPE in livestock and food, in order to identify potential (future) sources.

In general, resistance genes located on the chromosome are considered to pose with a lower risk of horizontal transfer to other bacteria than plasmid carried resistance.^17^ However, in the present study, targeted PCR experiments showed that *bla*_OXA-244_ gene in both *E. coli* isolates is mobilized by IS*1* elements, forming small circular elements that could integrate into a mobile genetic element such as a plasmid.^18^ Further research is required to fully understand the implication of this hypothetical route for spread of *bla*_OXA-244_.

Both OXA-244-positive *E. coli* isolates were identified with selective enrichment followed by PCR-screening and bacterial culturing as described above, whereas they remained undetected using the EURL-AR protocol. Their low carbapenem MIC-values resulted in poor or absent growth on ChromID^®^ OXA-48 and ChromID^®^ CARBA plates, consistent with earlier findings that this OXA-variant poses a challenge for routine detection.^1,19^

Our results demonstrate the added value of implementing a more sensitive screening method for detecting CPE with low-level resistance to carbapenems like OXA-244. In response, EURL-AR has amended the isolation protocol by recommending an alternative commercial medium to improve the detection of OXA-244.^7^ EFSA has also identified knowledge gaps and emphasizes the need to improve detection methods, conduct trace-back investigations and apply molecular typing to clarify transmission routes, including potential spread via workers and feed.^1^ As a result, EURL-AR is coordinating an EFSA-funded project involving EU Member States and EFTA countries to improve detection and source attribution of CPE in the food chain, with the aim to better understand the potential risk to public health.

## Supplementary Material

dkag155_Supplementary_Data

## Supplementary data

Table S1 is available as Supplementary data at *JAC* Online.

## References

[1] European Food Safety Authority . Occurrence and spread of carbapenemase-producing Enterobacterales (CPE) in the food chain in the EU/EFTA. Part 1:2025 update. EFSA J 2025; 23: 1-87. 10.2903/j.efsa.2025.9336

[2] European Medicines Agency . Categorisation of antibiotics in the European Union. https://www.ema.europa.eu/en/documents/report/categorisation-antibiotics-european-union-answer-request-european-commission-updating-scientific-advice-impact-public-health-animal-health-use-antibiotics-animals_en.pdf.

[3] European Union . Commission Implementing Regulation (EU) 2022/1255.38. https://eur-lex.europa.eu/legal-content/EN/TXT/?uri=CELEX%3A32022R1255.

[4] European Food Safety Authority | European Centre for Disease Prevention and Control . The European Union summary report on antimicrobial resistance in zoonotic and indicator bacteria from humans, animals and food in 2022–2023. https://efsa.onlinelibrary.wiley.com/doi/10.2903/j.efsa.2025.9237.

[5] de Greeff SC, Kolwijck E, Schoffelen AF, et al. NethMap 2024. Consumption of antimicrobial agents and antimicrobial resistance among medically important bacteria in the Netherlands in 2023/MARAN 2024. Monitoring of antimicrobial resistance and antibiotic usage in animals in the Netherlands in 2023. https://www.rivm.nl/publicaties/nethmap-2024-consumption-of-antimicrobial-agents-and-antimicrobial-resistance.

[6] Broens E. A decade of monitoring carbapenemase producing Enterobacterales in dogs and cats in the Netherlands, ESCMID Global 2025, Abstract P1830.

[7] EU Reference Laboratory for Antimicrobial Resistance . Isolation of ESBL-, AmpC- and carbapenemase-producing E. coli from caecal samples. https://www.food.dtu.dk/english/topics/antimicrobial-resistance/eurl-ar/protocols.

[8] Technical Committee: ISO/TC 212. ISO 20776-1, Susceptibility testing of infectious agents and evaluation of performance of antimicrobial susceptibility test devices Part 1: Broth micro-dilution reference method for testing the in vitro activity of antimicrobial agents against rapidly growing aerobic bacteria involved in infectious diseases; 2019. https://www.iso.org/standard/70464.html

[9] European Committee on Antimicrobial Susceptibility Testing . MIC and Inhibition zone diameter distributions of microorganisms without and with phenotypically evident resistance mechanisms MIC and inhibition zone diameter distributions. https://mic.eucast.org/.

[10] Hendrickx APA, Landman F, de Haan A, et al *Bla*(OXA-48)-like genome architecture among carbapenemase-producing *Escherichia coli* and *Klebsiella pneumoniae* in The Netherlands. Microb Genom. 2021;7:000512. 10.1099/mgen.0.000512 33961543 PMC8209719

[11] Dankittipong N, Stegeman JA, de Vos CJ et al Investigating a propagation of emerging carbapenemase-producing Enterobacteriaceae in Dutch broiler production pyramid through stochastic simulation. One Health 2024; 19:100945. 10.1016/j.onehlt.2024.100945 39670196 PMC11635706

[12] Biedrzycka M, Izdebski R, Gniadkowski M et al Several epidemic and multiple sporadic genotypes of OXA-244-producing *Escherichia coli* in Poland; predominance of the ST38 clone. Eur J Clin Microbiol Infect Dis 2024; 43: 2465–72. 10.1007/s10096-024-04954-0 39373812 PMC11608361

[13] Welker S, Boutin S, Miethke T et al Emergence of carbapenem-resistant ST131 Escherichia coli carrying *bla*(OXA-244) in Germany, 2019 to 2020. Euro Surveill 2020; 25:2001815. 10.2807/1560-7917.ES.2020.25.46.2001815 33213685 PMC7678038

[14] European Centre for Disease prevention and Control (ECDC) . Increase in OXA-244-producing Escherichia coli in the European Union/European Economic Area and the UK since 2013 – first update: European Centre for Disease prevention and Control (ECDC); 2021. https://www.ecdc.europa.eu/sites/default/files/documents/RRA-E-coli-OXA-244-producing-E-coli-EU-EEA-UK-since-2013.pdf

[15] Sadek M, Ortiz de la Rosa JM, Ramadan M et al Molecular characterization of extended-spectrum ss-lactamase producers, carbapenemase producers, polymyxin-resistant, and fosfomycin-resistant Enterobacterales among pigs from Egypt. J Glob Antimicrob Resist 2022; 30: 81–7. 10.1016/j.jgar.2022.05.022 35667645

[16] Peirano G, Pitout JDD. Rapidly spreading Enterobacterales with OXA-48-like carbapenemases. J Clin Microbiol 2025; 63:e0151524. 10.1128/jcm.01515-24 39760498 PMC11837536

[17] David S, Cohen V, Reuter S et al Integrated chromosomal and plasmid sequence analyses reveal diverse modes of carbapenemase gene spread among *Klebsiella pneumoniae*. Proc Natl Acad Sci U S A 2020; 117: 25043–54. 10.1073/pnas.2003407117 32968015 PMC7587227

[18] Pitout JDD, Peirano G, Kock MM et al The global ascendency of OXA-48-type carbapenemases. Clin Microbiol Rev 2019; 33:e00102-19. 10.1128/CMR.00102-19 31722889 PMC6860007

[19] Emeraud C, Biez L, Girlich D et al Screening of OXA-244 producers, a difficult-to-detect and emerging OXA-48 variant? J Antimicrob Chemother 2020; 75: 2120–3. 10.1093/jac/dkaa155 32363407

