# Supplementary material for: Description of OXA-244 carbapenemase-producing Escherichia coli in farm animals in The Netherlands, 2024
Source: J Antimicrob Chemother. 2026 May 15;81(6):dkag155. doi: 10.1093/jac/dkag155 (PMC13175976; doi:10.1093/jac/dkag155)
Supplement: dkag155_Supplementary_Data [file dkag155_supplementary_data.docx]

**Supplementary data**

Within the framework of the AMR monitoring in animals and food according to European legislation (Decision EU/2020/1729), caecal samples from broilers, slaughter pigs and veal calves are collected at slaughter throughout the year using a stratified sampling design to exclude seasonal effects. In addition to the compulsory monitoring of these animal sectors, faecal samples from dairy cattle are included voluntarily collected at farms. As such, approximately 1200 samples (300 per animal species) are analysed on a yearly base. As part of the mandatory AMR program (Decision EU/2020/1729), all faecal samples of broilers, slaughter pigs and veal calves are screened for the presence of CPE according to the most recent version of the laboratory protocol published by the EURL-AR protocol^6^ based on non-selective enrichment in Buffered Peptone Water (BPW), followed by culturing on commercially available selective chromogenic plates (ChromID® OXA-48 and ChromID® CARBA, BioMérieux). Since 2012, these samples are also screened for the presence of CPE using an additional method based on selective enrichment followed by PCR screening and subsequent culturing of PCR-positive samples. In more detail, from each sample 0.1 gram was transferred into a 3 mL BPW with 50 mg/L vancomycin and 0.25 mg/L ertapenem using a sterile a cotton swab. The next day pools of each of five enriched BPW samples were prepared by pipetting 200 μL of each BPW enrichment in a tube with 2 mL lysis buffer. These tubes were stored at room temperature pending analysis. DNA isolation was performed using an EasyMag robot (BioMérieux). DNA was tested in a set of two multiplex PCRs and a PCR for amplification control, all runs using SybrGreen chemistry (SensiFast SYBR Lo-Rox; GC BIOTECH) and melt-curve analysis on an ABI PRISM 3100 Genetic Analyzer (Applied Biosystems), targeting NDM, KPC, VIM, IMP, OXA-48, IMI and FRI carbapenemase genes.

DNA is tested in a set of 2 multiplex PCR’s and a PCR for amplification control, all run using SybrGreen chemistry (SensiFast SYBR Lo-Rox; GC BIOTECH) and melt-curve analysis on an ABI PRISM 3100 Genetic Analyzer (Applied Biosystems).

The first multiplex PCR targets NDM, KPC, VIM, IMP and OXA, the second multiplex PCR targets IMI and FRI/FLC. Primers are listed below.

Table S1. Oligonucleotides for detection of carbapenemase genes used in the study.

| **Primers** | | | | |
| --- | --- | --- | --- | --- |
| **Target** | **Primer name** | **Sequence (5’-3’)** | **Product size (bp)** | **References** |
| NDM | NDM-624-F | CCTGATCAAGGACAGCAAGG | 130 | In-house |
|  | NDM-754-R | CGGAATGGCTCATCACGA |  |  |
| KPC | KPC-1-F | ATGTCACTGTATCGCCRTCT | 950 | V. Schechner et.al., 2009; with in-house modification, doi: 10.1128/JCM.02368-08 |
|  | KPC-920-R | CTCAGTGCTCTACAGAAAACC |  |  |
| VIM | VIM-591-F | TTGTGCCGTTCATGAGTTGT | 180 | In-house |
|  | VIM-771-R | TTTGACAACGTTCGCTGTGT |  |  |
| IMP | IMP-307-F | GGAATAGAGTGGCTTAAYTCTC | 188 | M.J. Ellington, 2006; doi: 10.1093/jac/dkl481 |
|  | IMP-495-R | CCAAACYACTASGTTATCT |  |  |
| OXA | OXA-31-F | TTGGTGGCATCGATTATCGG | 743 | L. Poirel et.al., 2012; doi: 10.1128/AAC.48.1.15-22.2004 |
|  | OXA-774-R | GAGCACTTCTTTTGTGATGGC |  |  |
| IMI | IMI-F | CCTATCAGACATGGTTAAAGG | 127 | WFSR, personal communication |
|  | IMI-R | GCATAATCATTTGCCGTACCG |  |  |
| FRI/FLC | FRI-F | GGCAGCAATTCAGTACAGTGA | 129 | WFSR, personal communication |
|  | FRI-R | TCCCAACGATCAAGCCTGAA |  |  |
| AC | AC-F | GATCAGCTACGTGAGGTCCTAC | 145 | Deer et al., 2010; doi: 10.1111/j.1472-765X.2010.02804.x |
|  | AC-R | CTAACCTTCGTGATGAGCAATCG |  |  |

In case of a PCR-positive pooled sample, DNA isolation and subsequent PCR screening was performed on the individual samples followed by bacterial culturing of 100 µL of the identified PCR-positive sample on several selective media: MacConkey agar with 0.125 mg/L ertapenem, ChromID® CARBA agar and ChromID® OXA agar plates. After 16-20 h incubation, plates were visually checked for growth of CPE-suspected colonies. After pure culturing on blood agar plates, identification was performed with MALDI-TOF (MALDI Biotyper® Sirius System, Bruker) followed by antimicrobial susceptibility testing using broth microdilution in harmonised European antibiotic panels containing 14 different antibiotics (EUVSEC3, Sensititre®, Thermo Scientific) according to ISO standards^7^. Results were interpreted with epidemiological cut-off values (ECOFFs) from EUCAST ^8^ . Whole Genome Sequencing (WGS) was performed using Illumina MiSeq and Oxford Nanopore Technologies MinION. Hybrid assembly was performed using Unicycler v0.5.1. Detection of resistance genes, mobile genetic elements and plasmids were performed with ResFinder v4.4.2, MobileElementFinder v1.0.3 and PlasmidFinder v2.0.1, respectively. Whole-genome multilocus sequence typing (wgMLST) and cluster analysis for *E. coli* were performed in context of Dutch CPE surveillance WGS data as described previously. ^9^

Inverse PCR and sequencing experiments were performed to test for the potential mobilization of the *bla*_OXA-244_ gene facilitated by IS elements using outward-directed primers targeting the *bla*_OXA-244_ gene, OXA-244 Rev-outward 5’-AGAGCACAACTACGCCCTGT-3’ and OXA-244 Fw-outward 5’- TCGGTTGGGTTGAACTTGAT-3 with BioMix Red (Bioline).
